# Plasma Fibroblast Growth Factor 23 Concentration Is Increased and Predicts Mortality in Patients on the Liver-Transplant Waiting List

**DOI:** 10.1371/journal.pone.0066182

**Published:** 2013-06-25

**Authors:** Dominique Prié, Anne Forand, Claire Francoz, Caroline Elie, Isabelle Cohen, Marie Courbebaisse, Dominique Eladari, Didier Lebrec, François Durand, Gerard Friedlander

**Affiliations:** 1 Université Paris Descartes, Faculté de Médecine, INSERM U845, Hôpital Necker-Enfants Malades, AP-HP, Paris, France; 2 INSERM U845 Paris, France; 3 Service d’Hépatologie Unité de Soins intensifs, INSERM U773, Hôpital Beaujon, AP-HP, Clichy, France; 4 Université Paris Descartes, Faculté de Médecine, EA 4472, Service de Biostatistique et Informatique Médicale Hôpital Necker-Enfants Malades, AP-HP, Paris, France; 5 Université Paris Descartes, Faculté de Médecine, INSERM UMRS 872, Service des Explorations Fonctionnelles et Radio-isotopes, Hôpital Européen Georges Pompidou, AP-HP, Paris, France; 6 Université Paris Descartes, Faculté de Médecine, INSERM U845, Service des Explorations Fonctionnelles et Radio-isotopes, Hôpital Européen Georges Pompidou, AP-HP, Paris, France; University of Colorado School of Medicine, United States of America

## Abstract

High plasma fibroblast growth factor-23 (FGF23) concentration predicts the risk of death and poor outcomes in patients with chronic kidney disease or chronic heart failure. We checked if FGF23 concentration could be modified in patients with end stage liver disease (ESLD) and predict mortality. We measured plasma FGF23 in 200 patients with ESLD registered on a liver transplant waiting list between January 2005 and October 2008. We found that median plasma FGF23 concentration was above normal values in 63% of the patients. Increased FGF23 concentration was not explained by its classical determinants: hyperphosphataemia, increased calcitriol concentration or decreased renal function. FGF23 concentration correlated with the MELD score, serum sodium concentration, and GFR. Forty-six patients died before being transplanted and 135 underwent liver transplantation. We analyzed the prognostic value of FGF23 levels. Mortality was significantly associated with FGF23 levels, the MELD score, serum sodium concentration and glomerular filtration rate. On multivariate analyses only FGF23 concentration was associated with mortality. FGF23 levels were independent of the cause of the liver disease. To determine if the damaged liver can produce FGF23 we measured plasma FGF23 concentration and liver FGF23 mRNA expression in control and diethyl-nitrosamine (DEN)-treated mice. FGF23 plasma levels increased with the apparition of liver lesions in DEN-treated mice and that FGF23 mRNA expression, which was undetectable in the liver of control mice, markedly increased with the development of liver lesions. The correlation between FGF23 plasma concentration and FGF23 mRNA expression in DEN-treated mice suggests that FGF23 production by the liver accounts for the increased plasma FGF23 concentration. In conclusion chronic liver lesions can induce expression of FGF23 mRNA leading to increased FGF23 concentration, which is associated with a higher mortality in patients on a liver-transplant waiting list. In these patients FGF23 concentration was the best predictor of mortality.

## Introduction

The liver expresses several fibroblast growth factors including FGF1, FGF2, FGF19, FGF21, FGF23 [1], [2], [3], [4]. Expressions of FGF1 and FGF2 are increased during hepatic injury or fibrogenesis and FGF8, which is expressed during liver development, is up-regulated in human hepatocellular carcinoma and in hepatitis C virus associated cirrhosis [5], [6], [7], [8], [9]. Although FGF23 mRNA is detected in fetal and adult liver alteration of its expression in cirrhosis or in liver injuries has not been studied so far. The aim of this study was to determine if plasma FGF23 concentration could be modified in end stage liver disease and the consequences of these modifications. Fibroblast growth factor 23 (FGF23) is a circulating peptide whose role is to control phosphate homeostasis and calcitriol levels [10]. It can be cleaved between amino acids 176–179 into two smaller peptides. The enzyme responsible for FGF23 cleavage and its location remains to be identified. FGF23 mRNA is mainly expressed in bone cells and the liver [3], [4]. FGF23 inhibits renal phosphate reabsorption and renal phosphate transporter expression [10]. Infusion or overexpression of FGF23 in animals or in humans results in the inhibition of 1-alpha hydroxylase (CYP27B1) activity in the kidney and the reduction of serum calcitriol concentration [11], [12], [13], [14], [15], [16]. Physiological triggers of FGF23 synthesis are high blood phosphate and calcitriol concentrations [11], [17], [18], [19], [20], [21]. FGF23 concentration also increases early with the decline of renal function [22], [23]. FGF23 affinity for FGF receptors (FGFR) is low. At physiological concentration FGF23 action requires the presence at the cell surface of a FGFR (type 1, 3 or 4) and the protein named αKlotho whose expression is restricted to few tissues. However, when FGF23 concentration increases, as observed when renal function declines or in chronic heart failure, FGF23 can activate different signaling pathways that are Klotho-independent. Hence at high concentration FGF23 could stimulate cardiac hypertrophy even in the absence of αKlotho [24]. High FGF23 concentration has been associated with elevated mortality in patients with various stages of chronic kidney disease or chronic heart failure or in community even in the absence of alteration of renal function [25], [26], [27], [28], [29], [30], [31]. Plasma FGF23 levels also predict the risk of progression of chronic kidney disease: the higher FGF23 concentrations, the higher risk of decrease in renal function [32]. All these data suggest that FGF23 concentration could be a predictor of mortality or poor outcomes in various disorders. To determine if FGF23 plasma concentration is increased in patients with advanced liver disease and if it could be a marker of prognosis, we measured FGF23 plasma levels in patients on a waiting list for liver transplantation. The only treatment of end stage liver diseases is liver transplantation consequently it is important to have biomarkers related to adverse outcome to allocate liver from deceased donors. In many countries the allocation of livers from deceased donors for transplantation uses the Model for End-Stage Liver Disease (MELD) score. This score is based on objective laboratory tests: the international normalized ration (INR) for the prothrombin time and the total bilirubin concentration, which assess the severity of liver cell dysfunction, and the serum creatinine concentration as an estimation of renal function. MELD score ranges between 6 and 40 [33]. Higher MELD scores indicate more severe disease and correlate with the risk of death within 3 months on the waiting list. Introduction of MELD score in the allocation of liver grafts has decreased the mortality of patients in the waiting list, however, in a context of organ shortage, waiting list mortality remains a challenging issue and various prognostic markers have been tested to optimize allograft allocation. Hence, serum sodium concentration is also a predictive factor in patients with liver cirrhosis and its combination with the MELD score has ameliorated the prediction of mortality [34], [35], [36], [37]. Other objective factors may contribute to further improve the prevention of death in patients in the liver transplant waiting list. FGF23 concentration can be measured quite easily by enzyme link immunosorbent assay (ELISA) and should be enter in routine in many diagnosis laboratories in the next few years. In this context we measured FGF23 levels in patients with end-stage liver disease on a liver transplantation waiting list and found that FGF23 concentration was increased even in the absence of renal insufficiency and was associated with the risk of death on the waiting list. Using diethyl-nitrosamine (DEN)-treated mice we demonstrated that chronic lesion of the liver induced the synthesis of FGF23 the liver. The levels of FGF23 mRNA expression in the liver and plasma FGF23 concentration increased with the severity of the liver lesion in mice, which may explain that FGF23 was the best predictor of mortality in human.

## Methods

We measured FGF23 concentration and glomerular filtration rate (GFR) using reference methods in all patients eligible for a first liver transplantation in the Department of Hepatology at Beaujon hospital, Clichy, France, from January 2005 to October 2008. Patients with fulminant hepatitis, or hospitalized in intensive care units or in dialysis were not considered in the study. Patient eligibility for liver transplant was determined in accordance with the guidelines by the Agence de la Biomédecine. From March 2007 a national score including the MELD score was introduced for each patient to be registered on the waiting list. Patients were referred to the Department of Clinical Investigation at Necker-Enfants Malades Hospital, Paris France, to measure their renal function before being registered on the waiting list for liver transplantation. At this time FGF23 plasma concentration was measured using Immutopics c-terminal Elisa kits (Human FGF23 c-terminal Elisa kit, Immutopics International, San Clemente California USA). We also used Kainos intact FGF23 Elisa kit (Kainos Laboratories Japan) to measure plasma intact FGF23 in a subgroup of patients. As reported by other groups we found a good correlation between the FGF23 values obtained with these two methods (Figure S1). Consequently only the c-terminal kit (Immutopics Inc San Clemente CA), which measures both intact FGF23 and its carboxyl terminal by-product, was used to measure FGF23 concentration in all patients. For FGF23 plasma determination 5 ml of blood were drawn on EDTA and immediately centrifuged at 4°C. The supernatant was stored at −80°C and used for measurement within two weeks.

Plasma FGF23 concentration was also determined in patients without acute or chronic liver disease (γ-glutamyl transpeptidase, aspartate aminotransferase, alanine aminotransferase and alkaline phosphatase, INR within the normal ranges) who were referred to the Department Explorations Fonctionnelles at Necker-Enfants Malades hospital for the control of renal function from January 2005 to October 2008. These patients had either renal lithiasis, or renal insufficiency or were eligible in the absence of altered renal function for a treatment by cyclosporine for psoriasis.

Plasma FGF23 concentration and glomerular filtration rate were measured with the same procedures in all patients. Glomerular filtration rate was measured by two methods of reference: inulin and iohexol clearance. The patients were injected with inulin (Inutest 25% Serb Laboratoires, France) or iohexol (Omnipaque 300 GE Healthcare). Urine and blood samples were collected every hour for 5 hours for the measurements of inulin and iohexol. Inulin was used when an allergy to iohexol was suspected on the base of the patient’s medical record.

Biochemical parameters were measured using routine biochemistry laboratory methods.

The MELD score was calculated with the use of the standard formula [38]. The MELD score ranges from 6 to 40 with higher values indicating more severe disease.

### Experimental Models

Mice were injected intraperitonealy with a single dose of diethyl-nitrosamine (DEN) (20 µg/g diluted in PBS) between 10 and 15 days of age. Total RNA was isolated from the liver using NucleoSpin RNA II columns (Macherey Nagel). RT-PCR amplifications were performed using M-MLV (Invitrogen) according to the manufacturer’s instructions. Real time PCR was performed with intron-spanning specific primers (the sequences are available upon request) using SyBr green chemistry (Thermo scientific) on an ABI prism 7000 detection system. Expression levels were normalized for pinin expression in the same sample. Data were analyzed with the 2^−ΔΔCT^ method described by Livak and Schmittgen [39].

Plasma intact FGF23 concentrations were assessed using a commercial ELISA according to the manufacturer**’**s protocol (Kainos Laboratories Inc.).

### Ethic Statement

All the measurements performed in this study were included in the routine procedures of patient follow-up except for FGF23 measurements. This study complied with the French rules regarding research in humans. Patients provided informed written consents. The study was approved by the Institutional Review Board Hôpital Beaujon, Clichy, France.

Animal care and maintenance were provided through the University Paris Descartes accredited Animal Facility at Necker Faculty of Medicine (Paris). Mice were maintained on standard rodent laboratory chow (Special Diet Services, UK). All procedures were approved by the Animal Care and Use Committee of the University Paris Descartes.

### Statistical Analysis

As the variable FGF23 concentration was not normally distributed, we used its log transformation in the whole analysis. Quantitative variables were described using mean ± SD or median (range).

Correlations of continuous variables with FGF23 were assessed by the Pearson correlation coefficients. Student t-test was performed to compare FGF23 between genders.

Survival time was defined as the time from the date of FGF23 measurement to death on waiting list, transplantation or last follow-up. We used two methods to analyze the survival on the waiting list. The first one considers transplantation as a censure using Cox proportional hazard model [40]. Survival rates on the waiting list were estimated by the Kaplan-Meier method [41]. The second relies on the competing risk analysis, transplantation and death before transplantation being two competing events. This analysis relies on proportional hazards models fitted using the method of Fine and Gray [42]. Both models gave very similar results (data not shown) and only those obtained with the first method (univariate and multivariate Cox proportional hazard model with transplantation as a censure) are reported here.

All statistical analyses were performed using R software (http://cran.r-project.org). P values below 0.05 were considered statistically significant.

Data obtained from animals were analyzed with non-parametric tests as indicated in the legends of the figures.

## Results

### FGF23 Plasma Concentration in the Patients on the Liver-transplant Waiting List

We measured plasma FGF23 concentration in 200 patients with end stage liver disease at the time when they were considered as eligible for liver transplantation. The main characteristics of these patients are presented in Table 1. At registration on the waiting list the median MELD score was 13.5 (range 6 to 40) and the median serum sodium concentration was 137 mmol/L (range 122 to 146). Twenty six percent of the patients (51 patients) had hyponatremia (serum sodium concentration <135 mmol/L). Forty two percent (84 patients) had hepatocellular carcinoma. The median FGF23 concentration was 241 RU/ml (range 5 to 17620). Plasma FGF23 concentration was above normal value (120 RU/ml) in 63% of patients (126 patients). Since on physiological condition FGF23 production is stimulated by serum phosphate or calcitriol concentration we measured these two parameters at the same time that FGF23 concentration. The median phosphate serum concentration was 0.95 mmol/L (range 0.53 to 1.86) at the time of FGF23 concentration measurement and hyperphosphatemia (serum phosphate concentration above 1.40 mmol/L) was present in only 3 patients. Median plasma calcitriol concentration was 23 pg/ml (range 5 to 117) (Table 1) and was below the upper normal value (50 pg/ml) in 185 (92.5%) of the patients.

**Table 1:** Characteristics of the patients on the liver-transplant waiting list.

Number of patients	200
Gender n (%)	Males : 145 (72)
	Females : 55 (28)
Age years (median, min-max)	53 (20–69)
Causes of cirrhosis n (%)	
Alcohol	81 (40.5)
Virus	76 (38)
other	43 (21.5)
MELD score (median, min-max)	14 (6–40)
Sodium serum concentration mmol/L(median, min-max)	137 (122–146)
MELD-Na score (median, min-max)	16 (4–55)
Refractory ascites n (%)	60 (30)
FGF23 RU/ml (median, min-max)	241 (5–17620)
Normal value<120 RU/ml	
Phosphate mmol/L (median, min-max)	0.95 (0.53–1.86)
Normal values: 0.80–1.40 mmol/L	
Glomerular filtration rate ml/min(median, min-max)	93 (12–178)
Plasma calcitriol concentration pg/ml(median, min-max)	26 (5–117)
Normal values: 15–50 pg/ml	

Most patients (81%, 162 patients) had a measured GFR above the lower normal limit (60 ml/min).

Correlations between FGF23 levels and clinical or biological factors are presented on Table 2. FGF23 concentration did not differ with gender (5.55±1.51 in men vs 5.50±1.28 in women, p = 0.86) and did not correlate with age (rho = −0.08, p = 0.29). Plasma FGF23 concentration was not correlated with phosphate or ionized calcium serum concentration or fractional excretion of phosphate in urine (Table 2) but was inversely correlated with plasma calcitriol concentration.

**Table 2:** Correlation of FGF23 concentration.

Variable	Correlation*	P value
Gender	Male = 5.55±1.51	0.86
	Female = 5.50±1.28	
Age	−0.08	0.29
MELD score	0.30	<0.0001
MELD-Na score	0.39	<0.0001
GFR	−0.42	<0.0001
Serum sodium concentration	−0.35	<0.0001
Serum phosphate concentration	−0.02	0.82
Plasma calcitriol concentration	−0.22	0.002
Serum ionized calcium	−0.07	0.34

* correlation coefficients are indicated except for gender (mean ±sd).

**Figure 1:**
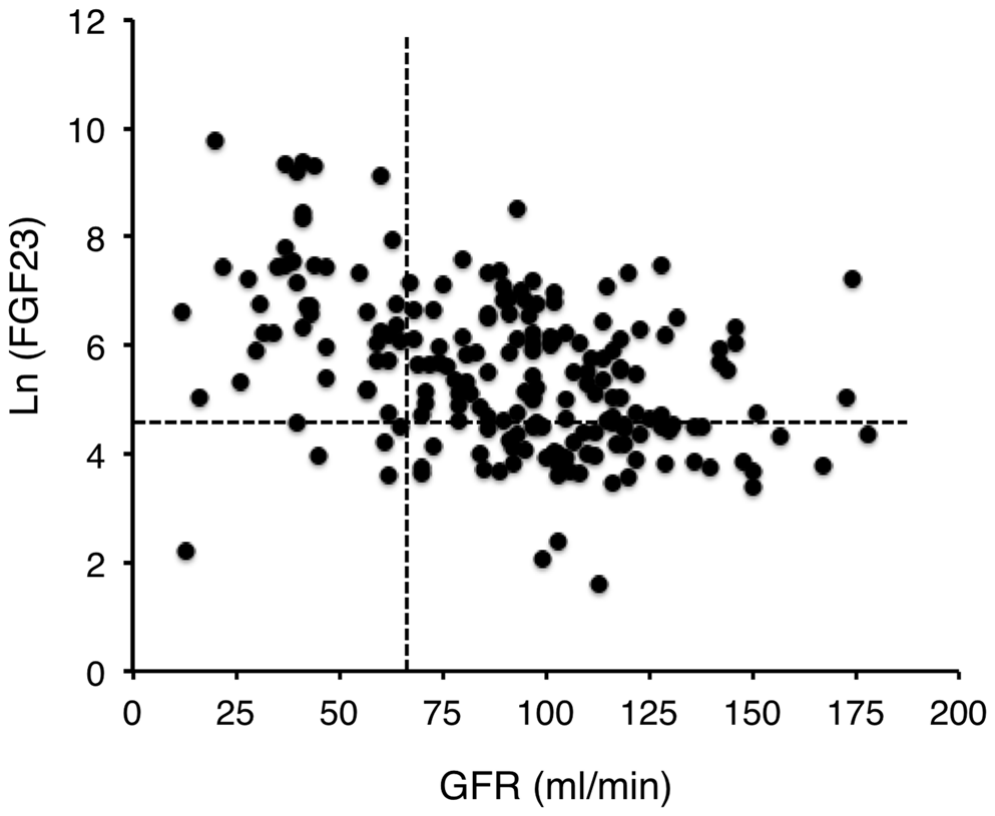
FGF23 concentration according to glomerular filtration rate (GFR) in patients with end-stage liver disease (ESLD). Dashed lines indicate upper and normal values of FGF23 and GFR respectively. FGF23 concentration was above normal values in most of subjects with ESLD and normal renal function (GFR>70 ml/min).

**Figure 2:**
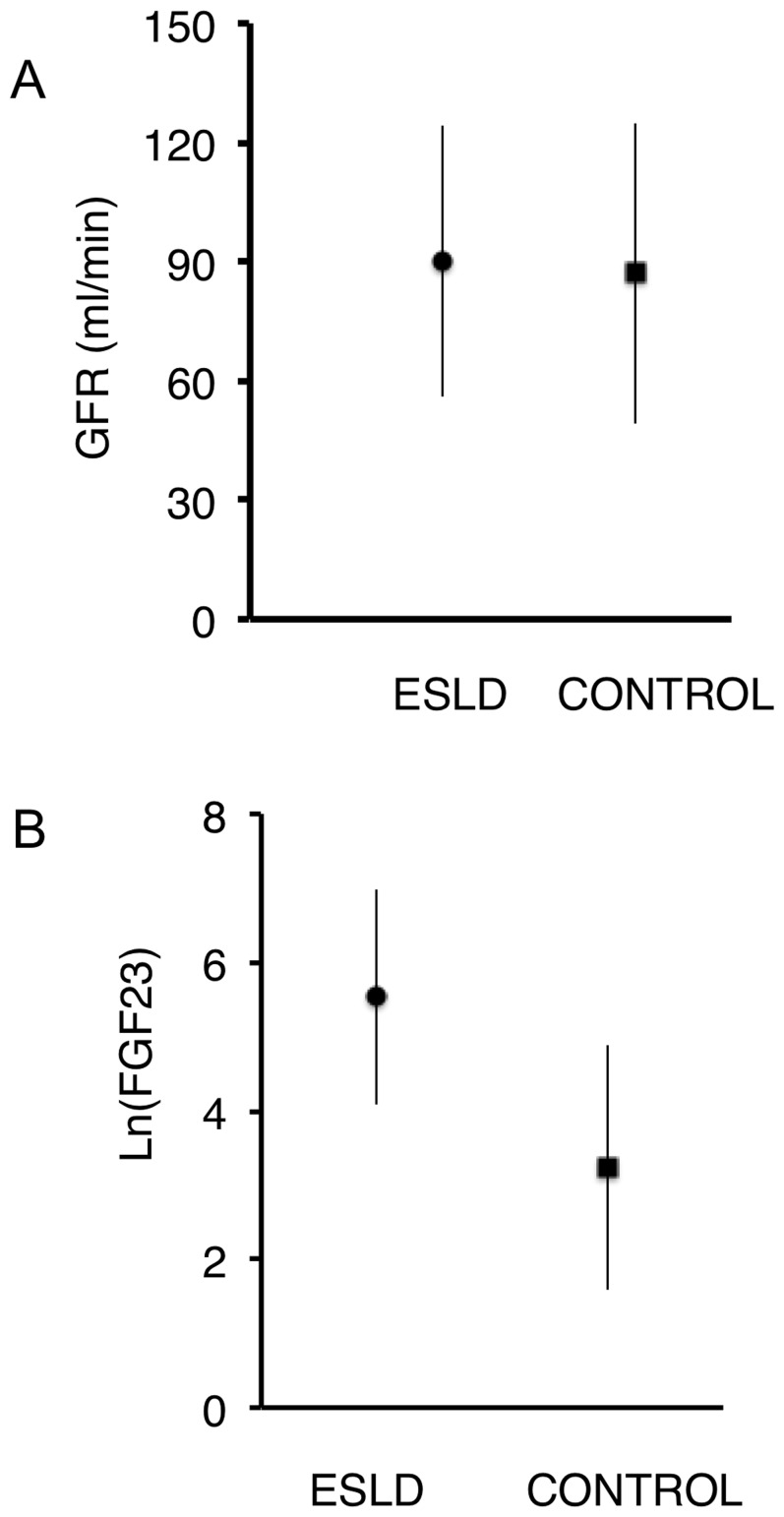
Comparison of FGF23 concentration between patients with end-stage liver disease (ESLD) or normal liver function (control). (A) Glomerular filtration rate (GFR) was not different between patients with ESLD and controls. (B) FGF23 concentration was significantly higher in patients with ESLD than in control subjects (p<0.0001). Results are mean ± SD.

As observed in patients with normal liver function, we found an inverse correlation between measured GFR and FGF23 (Table 2). However, FGF23 concentration was above the normal value in many patients with normal GFR values (figure 1): GFR was normal 81% of the patients on the waiting list, but FGF23 concentration was increased in 63% of them, suggesting that a decline in the GFR could not fully account for the increase in FGF23 levels. To confirm this point we compared FGF23 plasma concentration measured in the patients on the waiting list with that measured in 384 subjects (222 males, median age 50 years, range 16–85 years) without liver disease investigated in our department with the same procedure during the same period. GFR values were not different between these two groups (patients on waiting list 90±34 ml/min; control group 87±38 ml/min, p = 0.37. figure 2A). FGF23 plasma concentration was significantly higher in patients with cirrhosis (patients on waiting list Ln(FGF23) = 5.53±1.45; control group Ln(FGF23) = 3.23±1.65; mean ± sd, p<0.0001. figure 2B). This difference is still significant after adjusting for age and GFR (linear regression model). These results confirm that FGF23 plasma concentration was significantly higher in patients eligible for liver transplantation than in subjects without liver dysfunction and similar GFR.

FGF23 concentration correlated with MELD score and sodium serum concentration (Table 2) suggesting that it may be associated with the severity of the liver disease.

Plasma FGF23 concentration was not different in the patients with the viral or non-viral etiology of the liver disease (LnFGF23 mean ± SD: 5.44±1.45 viral hepatitis −; 5.70±1.42 viral hepatitis +; p = 0.21) but was significantly higher in patients with history of refractory ascites (LnFGF23 mean ± SD: 5.16±1.32 refractory ascites −; 6.40±1.37 refractory ascites +; p<0.001). The diagnosis of refractory ascites was made according to described criteria [43]. Plasma FGF23 concentration was significantly lower in subjects with hepatocarcinoma (LnFGF23 mean ± SD: 5.80±1.52 hepatocarcinoma −; 5.17±1.27 hepatocarcinoma +; p = 0.0026). Patients with hepatocarcinoma had less severe liver disease as suggested by better MELD-Na scores (13.9±5.4 vs 18.3±7.7, p<0.0001) and the lower frequency of refractory ascites (11.7% vs 88.3% p<0.0001).

### FGF23 Plasma Concentration and Mortality

Since the increase in FGF23 plasma concentration was significantly associated with two known prognostic markers of survival (MELD score and hyponatremia) in patients with liver diseases, we examined the association between FGF23 plasma levels and the risk of death in the patients on the transplant waiting list.

**Figure 3:**
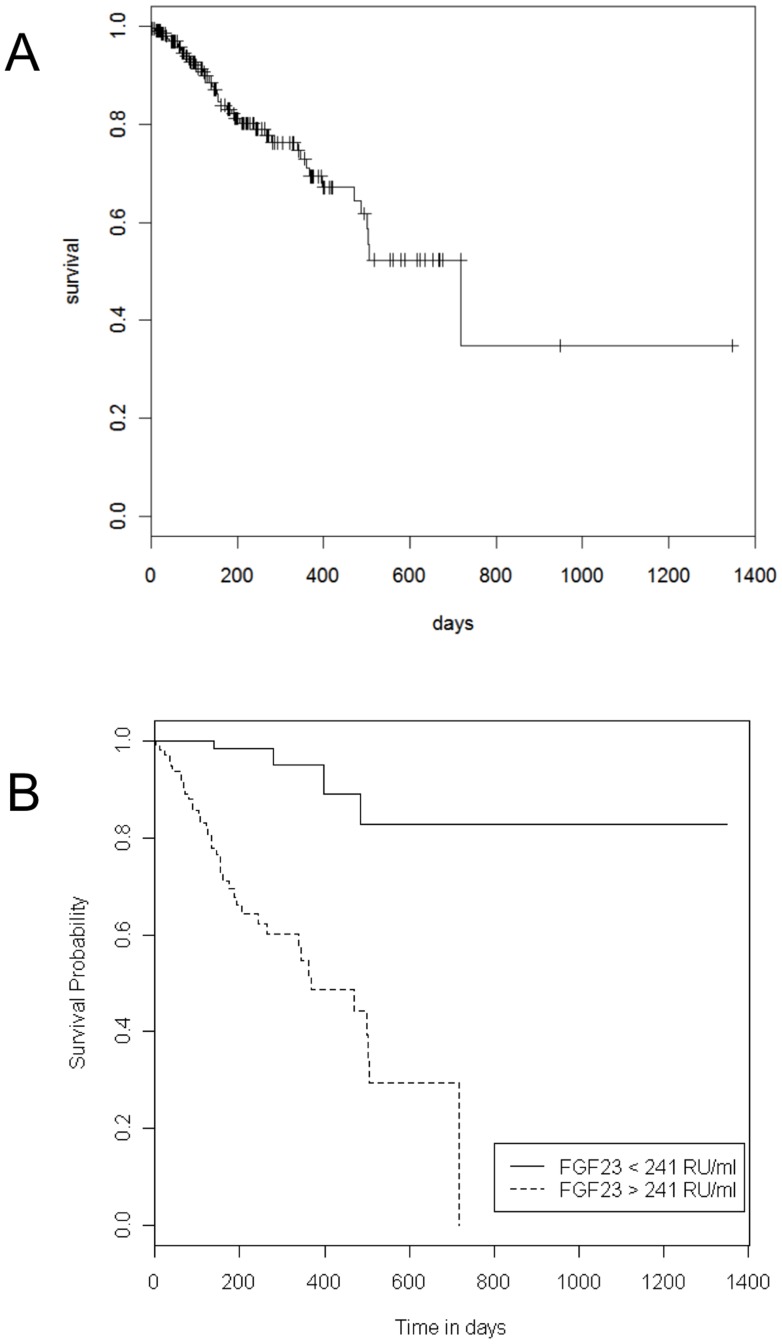
Patient survival according to plasma FGF23 concentration at the time of registration on the transplant waiting list. (A) Kaplan-Meier survival-plot for patients on the liver–transplant waiting list. (B) Patient survival in relation to plasma FGF23 concentration measured at the time of the registration on the transplant waiting list. Dashed line represents FGF23 concentration >241 RU/ml and plain line FGF23 concentration ≤241 RU/ml.

During the time of the study, 135 patients underwent liver transplantation, 43 died before being transplanted (22%) and 22 were still on the waiting list at the end of the study. During the study period, none of the patients included in this study were removed from the waiting list. Median follow-up was 201 days (range 2 to 1347). Kaplan-Meier survival curve is shown on figure 3A.

On univariate analysis (Table 3), the risk of death increased significantly with FGF23 plasma concentration, MELD and MELD-Na scores and decreased significantly with GFR and sodium serum concentration. Gender and age were not prognostic factors on the waiting list (Table 3).

**Table 3:** Univariate analysis of prognostic factors on the waiting list.

	Hazard ratio	CI 95%	P value
Gender	0.87	[0.45–1.68]	0.68
Age*	1.01	[0.97–1.04]	0.69
Ln(FGF23)*	2.03	[1.68–2.45]	<0.0001
MELD score*	1.03	[1.01–1.05]	0.04
GFR*	0.98	[0.97–0.99]	<0.0001
Serum sodium concentration*	0.84	[0.79–0.90]	<0.0001
MELD-Na score*	1.04	[1.02–1.07]	0.002
Viral hepatitis	0.84	[0.43–1.61]	0.59
Refractory ascites	2.89	[1.59–5.26]	0.001
Hepatocellular carcinoma	0.49	[0.25–0.97]	0.04

Analyses were performed with the use of a Cox proportional-hazard analysis considering transplant as a censor.

* HR for an increase of one unit of the variable.

GFR: glomerular filtration rate.

On multivariate analysis including FGF23, MELD-Na score and GFR, refractory ascites history and the presence of hepatocarcinoma, only FGF23 plasma concentration remained significantly associated with an increased risk of death (hazard ratio 2.21; 95% CI, 1.69 to 2.92, p<0.001) (Table 4).

**Table 4:** Multivariate analysis of prognostic factors on the waiting list.

	Hazard ratio	CI 95%	P value
Ln(FGF23)*	2.21	[1.67–2.93]	<0.0001
GFR*	1.00	[0.97–1.01]	0.67
MELD-Na score*	0.97	[0.94–1.01]	0.11
Refractory ascites	0.88	[0.40–1.94]	0.75
Hepatocellular carcinoma	0.75	[0.34–1.67]	0.48

Analyses were performed with the use of a Cox proportional-hazard analysis considering transplant as a censor.

* HR for an increase of one unit of the variable.

GFR: glomerular filtration rate.

Survival was markedly lower in patients with serum FGF23 concentration above median value (>241 RU/ml) (figure 3B).

### Production of FGF23 by the Liver

**Figure 4:**
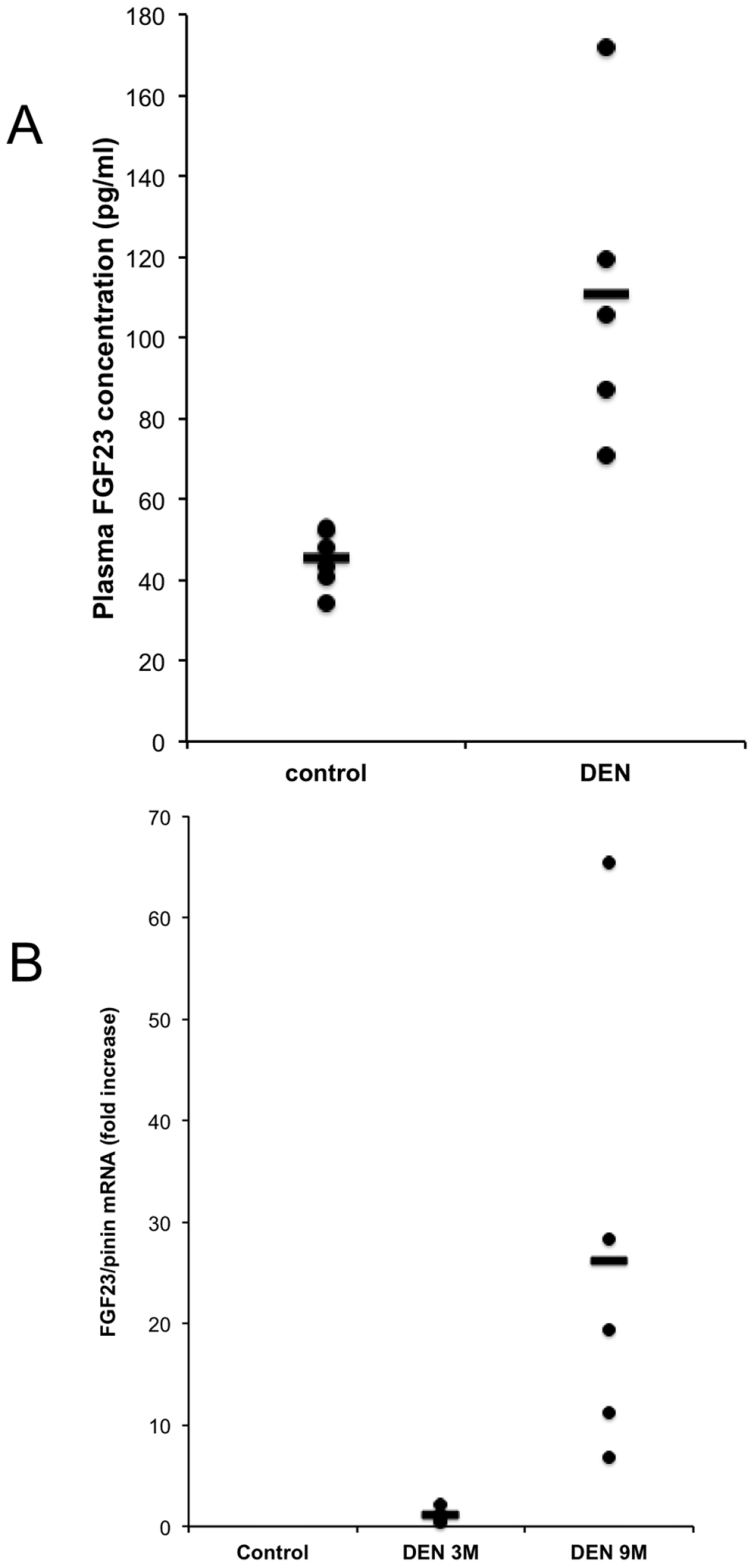
FGF23 plasma concentration and FGF23 mRNA level quantification in control and DEN- treated mice. (A) FGF23 plasma concentration in control mice (n = 6) and in DEN-treated mice (n = 6). The bars represent the means, and the circles the individual sample values. Samples were compared with the median test, p<0.005. (B) FGF23 mRNA expression was measured by qRT-PCR in the liver of control (untreated) mice and DEN-treated mice at 3 (n = 5) and 9 (n = 5) months after DEN injection. Since FGF23 mRNA was undetectable in control mice the fold of increase were expressed by comparison to 3 month DEN-treated mice. The bars represent the means, and the circles the individual sample values. Samples from DEN-treated mice were compared with the median test, p<0.005.

**Figure 5:**
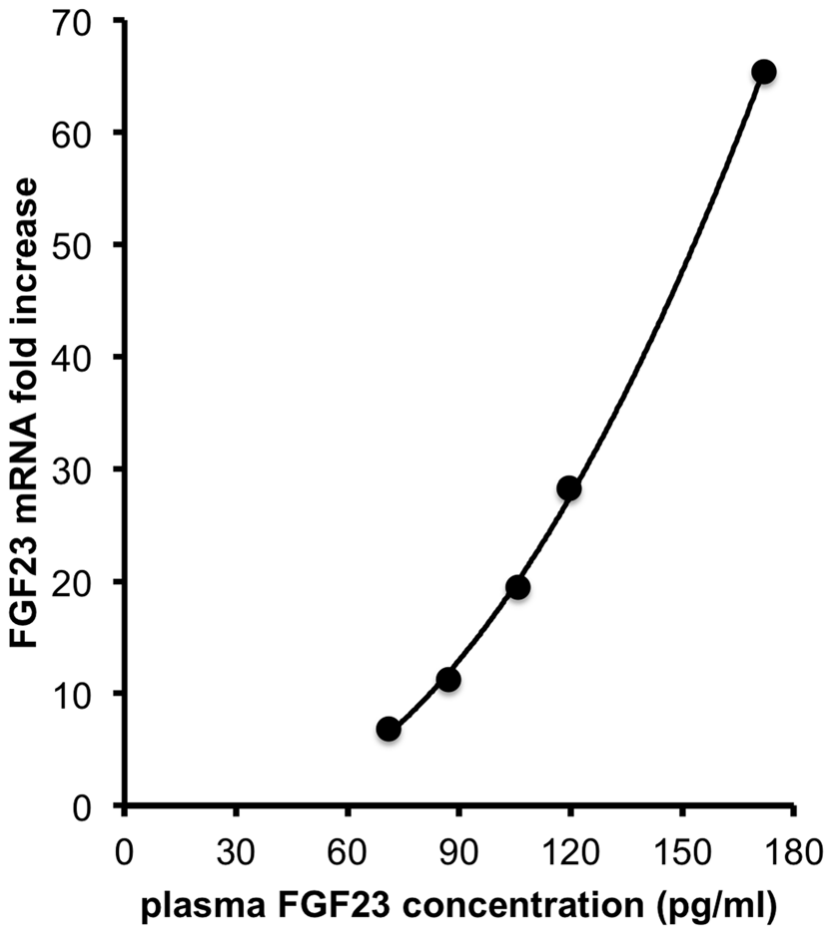
Correlation of plasma FGF23 concentration with liver FGF23 mRNA expression in DEN treated mice at month 9. Plasma FGF23 concentration and FGF23 mRNA expression were measured in 5 mice 9 month after a single injection of DEN. The best fitting was obtained with a second order polynomial curve (R^2^ = 0.9993). The best fitting equation was determined by GraphPad Prism 5 for mac.

Our results in human with ESLD suggested that plasma FGF23 increase could be induced by the severity of chronic liver lesions. To confirm this point we measured plasma FGF23 concentration in control mice and in mice that received one injection of diethyl-nitrosamine (DEN) and developed liver lesions. Nine months after DEN injection plasma FGF23 concentrations were significantly higher in DEN-treated mice (figure 4A) than in controls. To determine if production of FGF23 by the liver could participate to the increase in FGF23 concentration we measured by quantitative rtPCR FGF23 mRNA expression in the liver of control mice and DEN-treated mice 3 and 9 months after DEN injection. FGF23 mRNA was undetectable in control mice at any age but was present in the liver of all DEN-treated mice (figure 4B). Because liver lesions develop with time in DEN-treated mice we compared the expression of liver FGF23 mRNA at 3 and 9 months after DEN injection. Liver FGF23 mRNA expression significantly increased between month 3 and month 9 (figure 4B). To assess if the increase in FGF23 mRNA expression in the liver could account for the increase in plasma FGF23 concentration we plotted for each mouse the level of liver FGF23 mRNA expression against FGF23 plasma expression. The correlation between liver FGF23 mRNA and plasma FGF23 concentration fitted best with a second order polynomial (quadratic) curve (R^2^ = 0.9993) (Figure 5).

## Discussion

In this study we report for the first time that plasma FGF23 concentration is increased in patients with end stage liver disease and we show that FGF23 plasma levels predict the risk of death in patients on the liver-transplant waiting list. Multivariate analysis indicates that FGF23 concentration was the best predictor of the risk of mortality. Multivariate analysis indicates that FGF23 concentration is a predictor of mortality independent of renal function in patients on the liver-transplant waiting list.

The sample on the liver-transplant waiting list did not differ by its characteristic from those reported in other studies, regarding the distributions of age, MELD and MELD-Na score, serum sodium concentration, sex ratio, the causes of cirrhosis or the number of deaths during the 44 months of the study [44], [45], [46], [47], [48]. As reported in other studies we observed that the MELD and MELD-Na scores and serum sodium concentration were associated with an increased risk of death in our patients.

Univariate analyses using transplant as a censor or considering death and liver transplant as competing risks indicate that FGF23 concentration was markedly associated with patient survival. Indeed, an increase in each unit of logarithm of FGF23 doubles the risk of death. Survival curves show that FGF23 concentration above 240 RU/ml is markedly associated with an increased risk of death.

Multivariate analyses confirmed that plasma FGF23 concentration measured at the time of registration on the waiting list predicts the risk of death independently and better than the commonly used parameters including MELD-Na score, or the GFR value. Whether the combination of FGF23 concentration with the MELD-Na score may further improve the prediction of mortality in the waiting list remains to be determined.

We found an inverse correlation between FGF23 levels and circulating calcitriol concentration suggesting that FGF23 was biologically active. This correlation is in line with the results of the measurements made with the kit that specifically measures intact FGF23.

Several studies reported an association between FGF23 concentration and an increased risk of death, cardiac hypertrophy, heart failure, atrial fibrillation in different population including dialysis patients, patients with moderate alteration of kidney function, subjects with normal GFR and in subjects with chronic heart disease [29], [30], [31], [49], [50], [51], [52]. Our results show that high plasma FGF23 concentration are associated with an increased risk of death also in patients with ESLD on a transplantation waiting list. Recent data suggest that FGF23 may be directly responsible for the increased risk of death because of toxic effects when its concentration rises above physiological values [24]. Under physiological conditions FGF23 binds to its receptor made of a FGF receptor (FGFR type 1, 3 or 4) and the protein Klotho. Only cells that co-express a FGFR and Klotho are sensitive to FGF23 signaling. However when FGF23 plasma levels increase above physiological values FGF23 can exhibit off-target effects. It can stimulate FGFR in the absence of Klotho and trigger new signaling pathway in particular on cardiomyocytes [24]. This mechanism participate to the left ventricular hypertrophy and the increased risk of mortality associated with high FGF23 levels. FGF23 may similarly increase the risk of death in patients with end stage liver disease. However it is likely that FGF23 “off-target” effects are not restricted to the heart. FGF23 may also increase the sensitivity to infection. Indeed FGF23 inhibits calcitriol synthesis and enhances the expression of CYP24A1, the enzyme that degrades calcitriol and 25OH vitamin D. Calcitriol induces innate antimicrobial response, suppresses pro-inflammatory cytokine response via endocrine, paracrine and autocrine activity. Low calcitriol and 25 OH vitamin D concentrations have been associated with an increase risk of infection and an increased risk of death [53], [54].

The increase in FGF23 concentration was not triggered by the physiological stimuli of FGF23 production by bone cells. Indeed plasma calcitriol concentrations were below the upper normal range in most of the patients elevated FGF23. Similarly a decrease in renal function cannot account for the increase in FGF23 concentration since first most subjects with ESLD and elevated FGF23 concentration had normal renal function and second when matched for GFR with control subjects, ESLD patients had higher FGF23 concentrations. On physiological condition the main source of FGF23 is the osteocyte [3]. In some studies low levels of FGF23 mRNA have been detected in normal liver [3], [12], [55], [56]. We found that FGF23 mRNA was present in the liver of mouse fetuses (data not shown) but not in adults. Our results obtained in an animal model with DEN-induced chronic liver lesions support the view that chronic injury of liver cells induces a marked re-expression of FGF23 that can account for the increase in plasma FGF23 concentration as suggested by the correlation between FGF23 mRNA levels in the liver and plasma FGF23 concentration in DEN treated mice. The re-expression of FGF23 in the liver is independent of the presence of a virus or a hepatocarcinoma but seems to reflect the severity of liver dysfunction. We could not assess FGF23 mRNA expression in the liver of the patients because we did not have liver biopsies or explanted livers available for RNA extraction for the patients studied. Consequently we studied FGF23 mRNA expression in the liver of DEN-treated mice. We observed a correlation between FGF23 production and the severity of liver lesions. This is in line with the correlation we observed between FGF23 concentration and the MELD score whose two main components, INR and total bilirubin concentration, are related to liver cell functions.

In conclusion we report that plasma FGF23 concentration is increased in patients with end stage liver disease on a waiting list for liver transplantation and is markedly associated with an increased risk of mortality. Our results suggest that circulating FGF23 is due to FGF23 mRNA re-expression by liver cells during chronic liver lesions. In our patients in the liver-transplant waiting list FGF23 was the best predictor of survival. Additional multicentric studies enrolling a greater number of patients are needed to confirm our findings and validate that plasma FGF23 concentration, which can be easily measured using an ELISA, could be used as a new marker, alone or in combination with classical markers, to elaborate new rules to prioritize the allocation of liver grafts on the basis of the risk of death for the patients.

## Supporting Information

Figure S1 **Correlation between Log of FGF23 levels measured with the intact FGF23 assay (Kainos laboratories) and the C-terminal FGF23 assay (Immutopics).**
(TIF)
